# A hemodynamic analysis of energy loss in abdominal aortic aneurysm using three-dimension idealized model

**DOI:** 10.3389/fphys.2024.1330848

**Published:** 2024-01-19

**Authors:** Lulu Wang, Xudong Jiang, Kejia Zhang, Kai Chen, Peng Wu, Xiaoqiang Li

**Affiliations:** ^1^ Department of Vascular Surgery, Nanjing Drum Tower Hospital, Affiliated Hospital of Medical School, Nanjing University, Nanjing, China; ^2^ Artificial Organ Technology Laboratory, School of Mechanical and Electrical Engineering, Soochow University, Suzhou, China; ^3^ School of Mechanical Engineering, Jiangsu Key Laboratory for Design and Manufacture of Micro-Nano Biomedical Instruments, Southeast University, Nanjing, China

**Keywords:** abdominal aortic aneurysm, CFD, energy loss, thrombosis, rupture

## Abstract

**Objective:** The aim of this study is to perform specific hemodynamic simulations of idealized abdominal aortic aneurysm (AAA) models with different diameters, curvatures and eccentricities and evaluate the risk of thrombosis and aneurysm rupture.

**Methods:** Nine idealized AAA models with different diameters (3 cm or 5 cm), curvatures (0° or 30°) and eccentricities (centered on or tangent to the aorta), as well as a normal model, were constructed using commercial software (Solidworks; Dassault Systemes S.A, Suresnes, France). Hemodynamic simulations were conducted with the same time-varying volumetric flow rate extracted from the literature and 3-element Windkessel model (3 EWM) boundary conditions were applied at the aortic outlet. Several hemodynamic parameters such as time-averaged wall shear stress (TAWSS), oscillatory shear index (OSI), relative residence time (RRT), endothelial cell activation potential (ECAP) and energy loss (EL) were obtained to evaluate the risk of thrombosis and aneurysm rupture under different conditions.

**Results:** Simulation results showed that the proportion of low TAWSS region and high OSI region increases with the rising of aneurysm diameter, whereas decreases in the curvature and eccentric models of the corresponding diameters, with the 5 cm normal model having the largest low TAWSS region (68.5%) and high OSI region (40%). Similar to the results of TAWSS and OSI, the high ECAP and high RRT areas were largest in the 5 cm normal model, with the highest wall-averaged value (RRT: 5.18 s, ECAP: 4.36 Pa^−1^). Differently, the increase of aneurysm diameter, curvature, and eccentricity all lead to the increase of mean flow EL and turbulent EL, such that the highest mean flow EL (0.82 W · 10^−3^) and turbulent EL (1.72 W · 10^−3^) were observed in the eccentric 5 cm model with the bending angle of 30°.

**Conclusion:** Collectively, increases in aneurysm diameter, curvature, and eccentricity all raise mean flow EL and turbulent flow EL, which may aggravate the damage and disturbance of flow in aneurysm. In addition, it can be inferred by conventional parameters (TAWSS, OSI, RRT and ECAP) that the increase of aneurysm diameter may raise the risk of thrombosis, whereas the curvature and eccentricity appeared to have a protective effect against thrombosis.

## Introduction

Abdominal aortic aneurysm (AAA) is the most common type of aneurysm, resulting from permanent, limited dilatation of the arterial wall (Sakalihasan et al., 2018). The incidence of AAA is high, with a global prevalence of AAA of approximately 0.92% in people aged 30–79 years (Song et al., 2022). The most serious result of AAA is aneurysm rupture, with an overall mortality rate of 70%–90% (Assar and Zarins, 2009; Sakalihasan et al., 2018). Moreover, intraluminal thrombus (ILT) within AAA is another high-risk complication which has the risk of detachment, resulting in serious consequences such as acute lower extremity arterial embolization (Patel et al., 1994; Sakalihasan et al., 2018; Chen et al., 2023). Thus, it is necessary to evaluate the risk of thrombosis and rupture, and to intervene in advance.

The current clinical criteria for surgical intervention in AAA are based on the diameter of aneurysm, with the limitation that cannot explain the aneurysm rupture that occurs below the diameter threshold (Cronenwett et al., 1985; Nicholls et al., 1998). In addition, the variation in the geometry of AAA, such as eccentricity and curvature of the aneurysm, it may influence the progression of the aneurysm (Teng et al., 2022). The influence of these morphological characteristics on the risk of aneurysm thrombosis and rupture is usually associated with abnormal hemodynamic patterns within the aorta, and reflux and turbulent blood flow within the aneurysm has been reported to cause additional stresses on the wall and increase the rate of wall dilatation (Berguer et al., 2006; Khanafer et al., 2007).

Over the last decade, computational fluid dynamics (CFD) has been widely used to evaluate the hemodynamics of aortic disease (DAncona et al., 2014; Shang et al., 2015; Li et al., 2020), and has been well studied in assessing the risk of thrombosis and rupture of aneurysms. Di Achille et al. (Di Achille et al., 2014) assessed the risk of thrombosis in abdominal aortic aneurysms by using endothelial cell activation potential (ECAP), and found the metric was highly associated with thrombus formation and endothelial sensitivity. Relative residence time (RRT) represents the residence time of particles carried by the blood near the wall, and low RRT environment could reduce the risk of atherogenesis and thrombus formation and development (Zhan et al., 2022). Low wall shear stress (WSS) is thought to be more accurate than maximum diameter in predicting AAA rupture. Some studies have shown that rupture sites of AAA usually occur in areas of recirculation of blood flow with low WSS in the lumen (Boyd et al., 2016; Roux et al., 2020). In addition, the combination of low time-averaged wall shear stress (TAWSS) and high oscillatory shear index (OSI) may lead to intravascular abnormalities, which are also associated with aneurysm growth and rupture (Zambrano et al., 2016; Tzirakis et al., 2017). Although many studies have been performed, there are relatively few studies on the changing patterns of hemodynamic parameters of aneurysms with different morphological characteristics (e.g., aneurysm diameters, curvatures, and eccentricities). Moreover, in serval studies, the prediction parameters for thrombosis and rupture were ambiguous or even contradictory (Lozowy et al., 2017; Zambrano et al., 2022; Liang et al., 2023). Based on the above, there is an urgent need to propose other clinically meaningful metrics for predicting the progression of AAA and the risk of aneurysm rupture. Energy loss (EL), a novel biomechanical parameter used to describe the energy absorbed by the aortic wall, has been used to assess the risk of valvular stenosis (Pibarot et al., 2013; Shan et al., 2022) and aortic aneurysm dissection (Tang et al., 2021), and hemolysis in cardiac assistive devices (Torner et al., 2019), but has not yet commonly used in the assessment of aneurysms. Recently, Qiao et al. (Qiao et al., 2022) analyzed different types of EL by component quantification and pointed out its potential in evaluating aneurysm rupture; however, the study only compared the difference of EL in aneurysms with those in healthy aortas, and did not focus on the differences in EL between aneurysms with different geometries. Exploring the possible changing patterns of EL in aneurysms with different morphological characteristics, which may further enhance the credibility of EL as a novel predictor of the initiation and development of aneurysms.

Therefore, in this paper, hemodynamics simulations were performed on three-dimensional idealized abdominal aortic aneurysm models with different diameters, curvatures and eccentricities. Several hemodynamic parameters such as TAWSS, OSI, RRT and ECAP were used to access the risk of thrombosis and location of rupture in various type of aneurysms. In addition, the component quantification of EL was employed in this study as a supplementary metric to predict the potential of aneurysm rupture. This study may provide a novel approach to evaluate clinical interventions for abdominal aortic aneurysms.

## Materials and methods

### Idealized models

**FIGURE 1:**
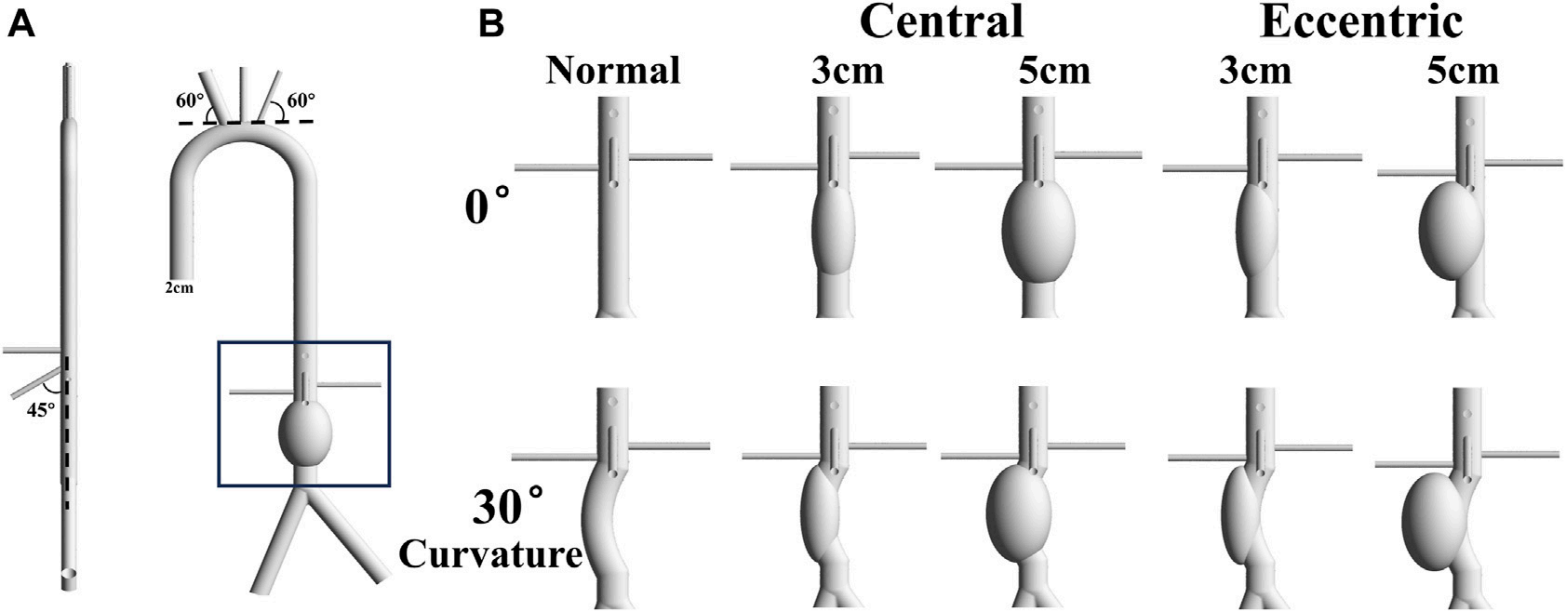
Idealized Model: **(A)** The normal idealized abdominal aortic aneurysm model with 5 cm diameter, and the diameter of aorta is 2 cm. The angle between the brachiocephalic trunk and the tangent of the aortic arch is 60°, and the angle between the superior mesenteric artery and the centerline of the aorta is 45°. The left renal artery is about a centimeter higher than the right renal artery; **(B)** 10 models include normal, central 3cm, central 5cm, eccentric 3 cm and eccentric 5 cm with the bending angle of 0 or 30°.

The normal idealized AAA model referenced from the present study (Qiao et al., 2022) was constructed using commercial software (Solidworks; Dassault Systemes S.A, Suresnes, France), and the aneurysm was below the level of renal arteries, as shown in Figure 1A. The diameter of the aneurysm was set to 3 cm and 5cm, representing the minimum size of the aneurysm and the minimum size that requiring surgical treatment, respectively. In order to investigate the effect of arterial curvature on aneurysms, the bending angle of the curvature model was set at 30°. In addition, to explore the effect of aneurysm eccentricity, the eccentric model was set as the aneurysm was tangent to the aorta. The artery structure at the inlet has an diameter of 20 mm, which is the typical size of a healthy aortic structure (Salman et al., 2019). Other parameters of idealized models, such as branch diameter, branch position, and branch angle, were absorbed from anatomical data or other literature (Kaewchoothong et al., 2022; Qiao et al., 2022). Therefore, 10 idealized model were conducted hemodynamic simulation in this study, as shown in Figure 1B. The diameters of aorta inlet and branch outlet were shown in Table1.

**TABLE 1:** Diameters of aorta inlet and branch outlet.

	BCT	LCA	LSA	CA	SMA	LRA	RRA	RIA	LIA
D (mm)	12	6.5	4	7	6.5	5	5	16	16

Note: BCT: brachiocephalic trunk. LCA: left carotid artery. LSA: left subclavian artery. LRA/RRA: right/left renal artery. CA: celiac artery. SMA: superior mesenteric artery. LIA/RIA: left/right iliac artery.

### Mesh and boundary conditions

**FIGURE 2:**
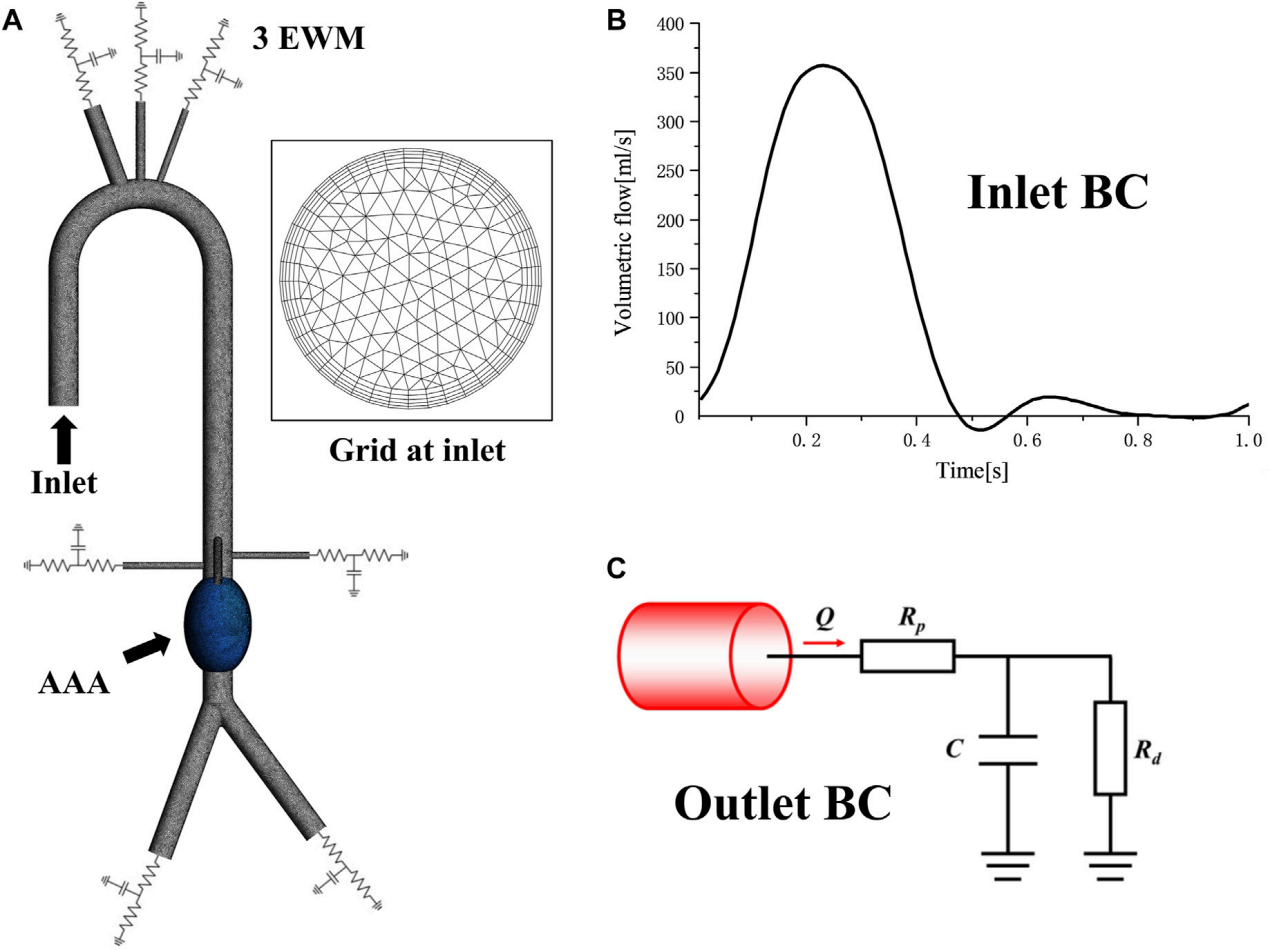
Mesh and boundary condition: **(A)** Computational domain mesh of normal 5 cm central model, showing domain division, local details and 5 prism layers grids at inlet; **(B)**The inlet velocity profile; **(C)** The Windkessel RCR outlet boundary conditions.

The purpose of this study was to investigate the hemodynamic changes and energy loss in abdominal aortic aneurysms under different conditions, thus the aneurysm was divided separately. All the inlets and outlets were extended to suppress the computational instability which may arise due to backflows. Structured grids of 2.65–3.26 million elements were generated by using commercial software Ansys Meshing (Ansys, Inc, Canonsburg, PA, USA) for the 10 models. Five grid layers were added to all the arterial walls to properly resolve the boundary layer to guarantee accuracy in hemodynamic parameters predictions, such as TAWSS, OSI, RRT, ECAP and EL, as shown in Figure 2A.

As shown in Figures 2A, B time-varying volumetric flow rate extracted from the literature was applied at the inlet with a period of 1s (Cheng et al., 2013). Windkessel RCR boundary conditions were applied at the aortic outlet, which describes the relationship between pressure and flowrate based on the analogy between electrical circuits and the cardiovascular system (Menichini and Xu, 2016; Pirola et al., 2017). The parameters of the 3-element Windkessel model (3 EWM) were calculated following our previous study (Jiang et al., 2022), and the converged parameter values were obtained through multiple rounds of iterative calculation, as shown in Table 2. All the walls were assumed to be rigid with no slip conditions.

**TABLE 2:** Values of the final 3 EWM parameters.

	BCT	LCA	LSA	CA	SMA	LRA	RRA	RIA	LIA
Rp (10^7^ Pa s·m^-3^)	0.4349	0.3152	3.0696	3.3982	0.5160	0.9350	48.2444	13.5101	17.9058
Rd (10^8^ Pa s·m^-3^)	11.5561	28.6869	28.3813	19.2580	31.5900	33.6934	62.3868	8.8715	9.6132
C (10^−10^ Pa^−1^ m^3^)	15.4316	7.9062	6.9682	9.1337	5.6571	5.2979	2.6632	17.5104	15.6965

Note: BCT: brachiocephalic trunk. LCA: left carotid artery. LSA: left subclavian artery. LRA/RRA: right/left renal artery. CA: celiac artery. SMA: superior mesenteric artery. LIA/RIA: left/right iliac artery.

### Hemodynamic parameters

TAWSS is commonly used as an indicator to assess the risk of thrombosis and rupture in arterial system, which is defined as:

$$TAWSS=\frac{1}{T}\int_{0}^{T}\left|WSS\right|dT$$ (1)

OSI is a dimensionless parameter originally used to identify atherosclerotic regions, which is defined as:

$$OSI=0.5\times \left[1-\frac{\left|\int_{0}^{T}WSSdT\right|}{\int_{0}^{T}\left|WSS\right|dT}\right]$$ (2)

ECAP is a parameter proposed by Di Achille et al. (Di Achille et al., 2014) to assess the risk of thrombosis in abdominal aortic aneurysms, which is defined as:

$$ECAP=\frac{OSI}{TAWSS}$$ (3)

RRT is a parameter to estimate the relative duration that blood resides close to the wall through a combination of TAWSS and OSI, which is defined as:

$$RRT=\frac{1}{\left(1-2OSI\right)TAWSS}$$ (4)

In addition, the values of TAWSS, RRT and ECAP in this paper were all wall-averaged values.

### Component quantification of energy loss

The total EL in the aorta can be considered as a hydraulic loss and can be calculated by the volume integral of the energy dissipation (Jiang et al., 2022), which is defined as:

$$W_{f}=\int_{V}\rho \varepsilon_{tot}dV$$ (5)

In turbulence, energy dissipation, the total EL of flow, occurs over the entire range of space and time motion. One of the commonly used analytical methods for turbulence analysis is to analyze the flow in a time statistical framework. The total energy dissipation rate $\varepsilon_{tot}$ can be statistically divided into two parts: mean flow EL $\varepsilon_{mean}$ and turbulence EL $\varepsilon_{turb}$ , which is defined as:

$$\varepsilon_{tot}=\varepsilon_{mean}+<\varepsilon_{turb}>=2\nu <S_{ij}><S_{ij}>+2\nu <S_{ij}^{\prime }S_{ij}^{\prime }>.$$ (6)

Mean flow EL $\varepsilon_{mean}$ represents the viscous dissipation of mean flow per unit mass of fluid in unit time, corresponding to the hydraulic loss due to the time-average velocity gradient. For strict laminar flow, average flow energy dissipation is the only source of EL. Turbulence EL $\varepsilon_{turb}$ represents the viscous dissipation per unit mass of fluid in unit time in the process of pulsating deformation. Due to the high resolution of the spatial and temporal, most turbulence simulation methods use a combination of analytical and simulation methods:

$$<\varepsilon_{turb}>=<\varepsilon_{turb,res}>+<\varepsilon_{turb,\mathrm{mod}}>=2\nu <S_{ij}^{\prime }S_{ij}^{\prime }>+2<\nu_{t}S_{ij}S_{ij}>.$$ (7)

Thus, total EL also can be expressed as:

$$W_{f}^{\prime }=\int_{V}\rho \left(\varepsilon_{mean}+<\varepsilon_{turb,res}>+<\varepsilon_{turb,\mathrm{mod}}>\right)dV.$$ (8)

Expression (Chen et al., 2023) and Expression (Teng et al., 2022) are the indirect and direct solutions of total EL in the aorta, respectively. The two parts should have the same value.

In general, mean flow EL occurred more on the wall of blood vessels, which was highly correlated with vascular damage, while turbulent dissipation was more likely to occur in the disordered flow field, which was associated with the instability of flow pattern, indicating the risk of aneurysm rupture.

### Numerical simulation

In this study, all simulations were transient and conducted using the commercial software Ansys Fluent (Ansys, Inc, Canonsburg, PA, USA). Blood was regarded as incompressible Newtonian fluids, with density of 1055 kg/m^3^, and dynamic viscosity of 3.5 × 10^−3^ Pa·s, and the flow was assumed to be turbulent using the RNG model. A second-order implicit backward Euler scheme was chosen for temporal discretization, with a fixed time-step of 10 m so that per cardiac cycle was resolved using 100 time steps. Maximum 50 sub-iterations were used for each physical time step, and the maximum RMS residual was set to 10^−5^ as a convergence criterion. Unsteady simulations were carried out for about 10 cardiac cycles to get statistically converged flow field, followed by another 10 cardiac cycles to get hemodynamic parameters such as TAWSS, RRT and so on. The EL needed to be calculated separately on this basis by compiling user defined function. All computations were carried out on a 192-core cluster equipped with 16 Intel Xeon E5-2680 v3 CPUs. The simulations normally converged within 10 h.

## Results

### TAWSS&OSI

**FIGURE 3:**
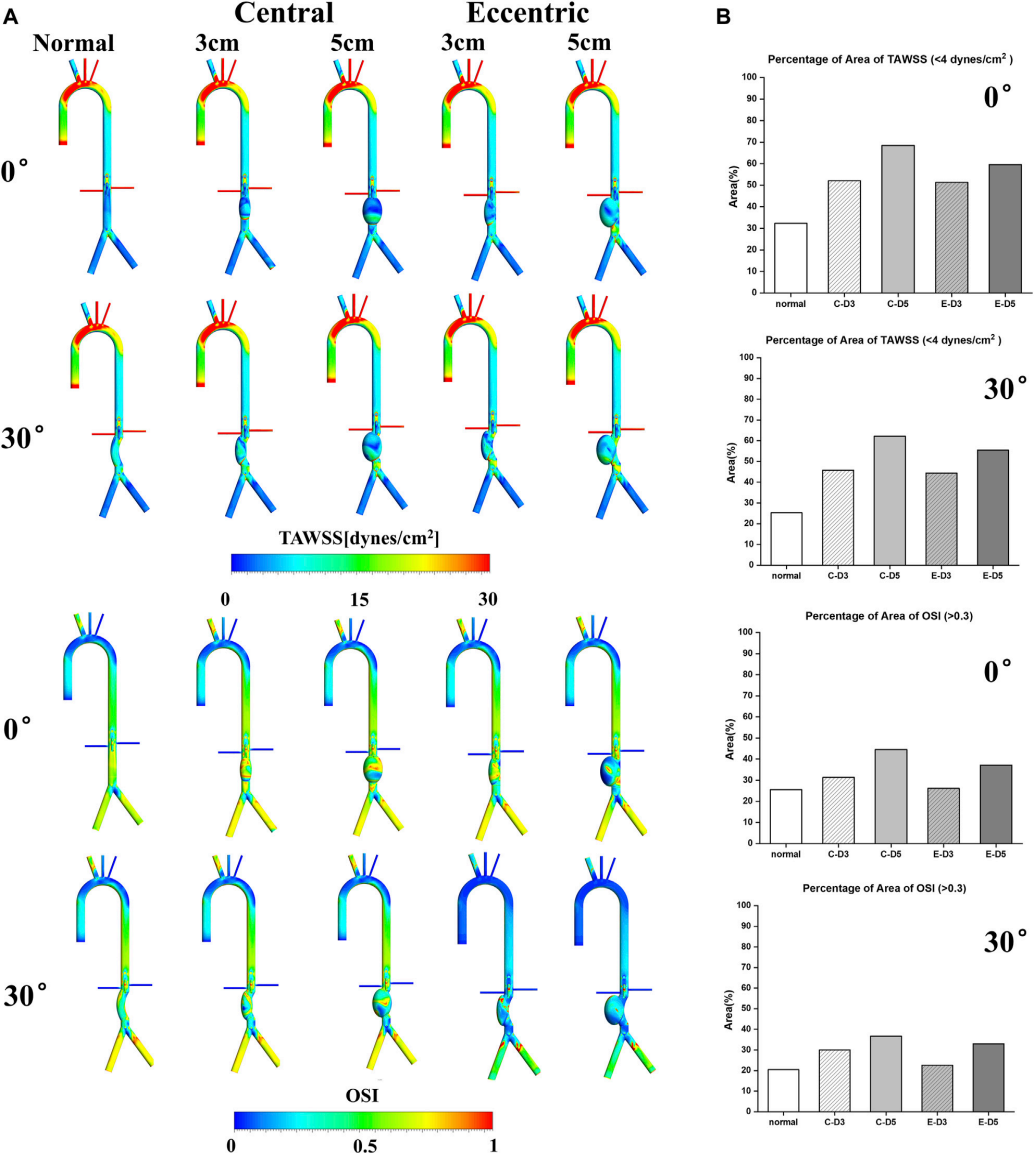
TAWSS&OSI: **(A)** TAWSS&OSI contours, the TAWSS on aneurysm wall was generally low, with the low TAWSS area concentrated in the upper part of the aneurysm. In the eccentric model and the curvature model, the low TAWSS region also appeared in the lateral edge of the aneurysm. Similarly, areas with high OSI had a similar distribution to those with low TAWSS, the value of OSI on aneurysm is generally high; **(B)** The percentage of area of low TAWSS and high OSI on aneurysm, the C represents central model and the E represents eccentric model.

TAWSS and OSI are conventional hemodynamic parameters in the arterial system, which play a significant role in predicting the thrombosis risk in aneurysm and the location of aneurysm rupture. As shown in Figure 3A, the aortic arch was the region with large TAWSS, while the TAWSS on aneurysm wall was generally low, with the low TAWSS area concentrated in the upper part of the aneurysm. In the eccentric model and the curvature model, the low TAWSS region also appeared in the lateral edge of the aneurysm, which means that ruptures tend to occur in these areas. It can be found from the contours that the area of low TAWSS in 5 cm models were significantly more than that in 3 cm models, and the area of low TAWSS in both the eccentric model and the curvature model was lower than that in the normal model with the same aneurysm diameter. Similarly, areas with high OSI had a similar distribution to those with low TAWSS, the value of OSI on aneurysm is generally high.

It is generally believed that region with low TAWSS (<4 dyn/cm^2^) and high OSI (>0.3) prone to thrombosis (Cao et al., 2020). The percentage of area of low TAWSS and high OSI on aneurysm were collected and shown in Figure 3B. It can be observed that with the increase of aneurysm diameter, the area ratio of high OSI and low TAWSS region also increases, and the proportion in the curvature model and the eccentric model was significantly lower than that in the normal model, especially in the eccentric 3 cm model with a bending angle of 30%, The percentage of area of high OSI was 22.6%, which was even lower than the normal model (25.5%). The proportion of low TAWSS region (55.42%) and high OSI region (33%) in the 5 cm eccentric model with a bending angle of 30° were similar to that of the normal 3 cm model, in which the above two values were 52.1% and 31.3%, respectively. There was the highest proportion in the normal 5 cm model, with the low TAWSS region reaching 68.5% and the high OSI region reaching more than 40%. To sum up, larger aneurysms may more likely to thrombosis, and the curvature and eccentricity seem to prevent thrombosis.

### RRT&ECAP

**FIGURE 4:**
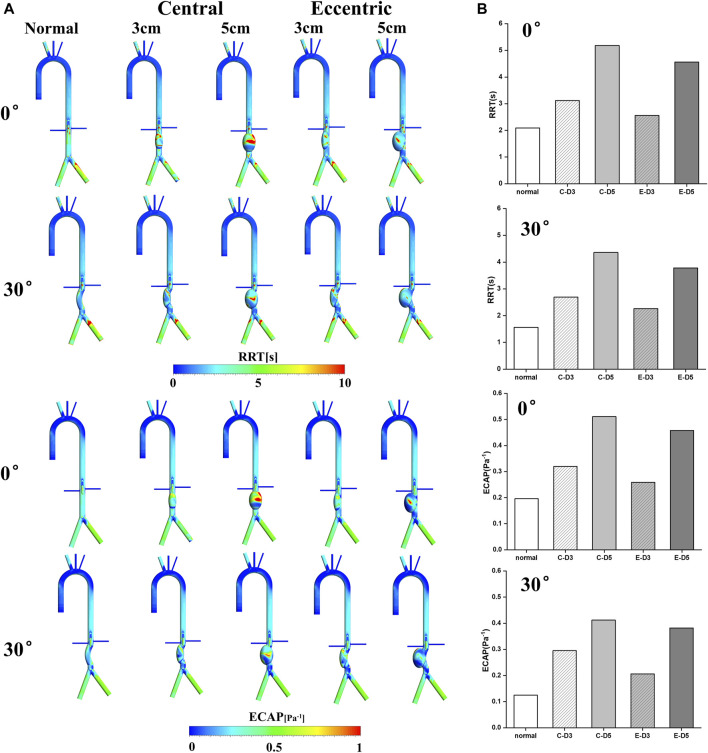
RRT&ECAP: **(A)** RRT&ECAP contours; **(B)** the wall-averaged RRT and ECAP in the aneurysm, the C represents central model and the E represents eccentric model.

In recent years, RRT and ECAP have been widely used to evaluate the thrombosis in the AAA, FL of aortic dissection and the auricular appendix. As shown in Figure 4A, it can be observed that RRT and ECAP had similar distributions. Consistent with the low TAWSS and high OSI regions mentioned above, RRT and ECAP on the aneurysm were significantly higher than those in other locations, further proving the high risk of thrombosis in the AAA. Especially in the normal 5 cm model, there was the largest area of high ECAP and high RRT on the aneurysm.

The wall-averaged RRT and ECAP were shown in Figure 4B. It can be observed that the 3 cm model RRT and ECAP were significantly lower compared with the 5 cm model, and the curvature model and eccentric model also had a lower value of RRT and ECAP than the normal model on aneurysm. The normal model with the bending angle of 30° showed the lowest wall-averaged value, in which RRT was 1.56s and ECAP was 0.125Pa^−1^. On the contrary, the highest value was in the normal 5 cm model, with the RRT reaching 5.18s and the ECAP reaching 4.36 Pa^−1^. It was worth noting that, similar to the results of OSI and TAWSS, the curvature and eccentricity seemed averse to the thrombosis in aneurysm.

### Energy loss

**FIGURE 5:**
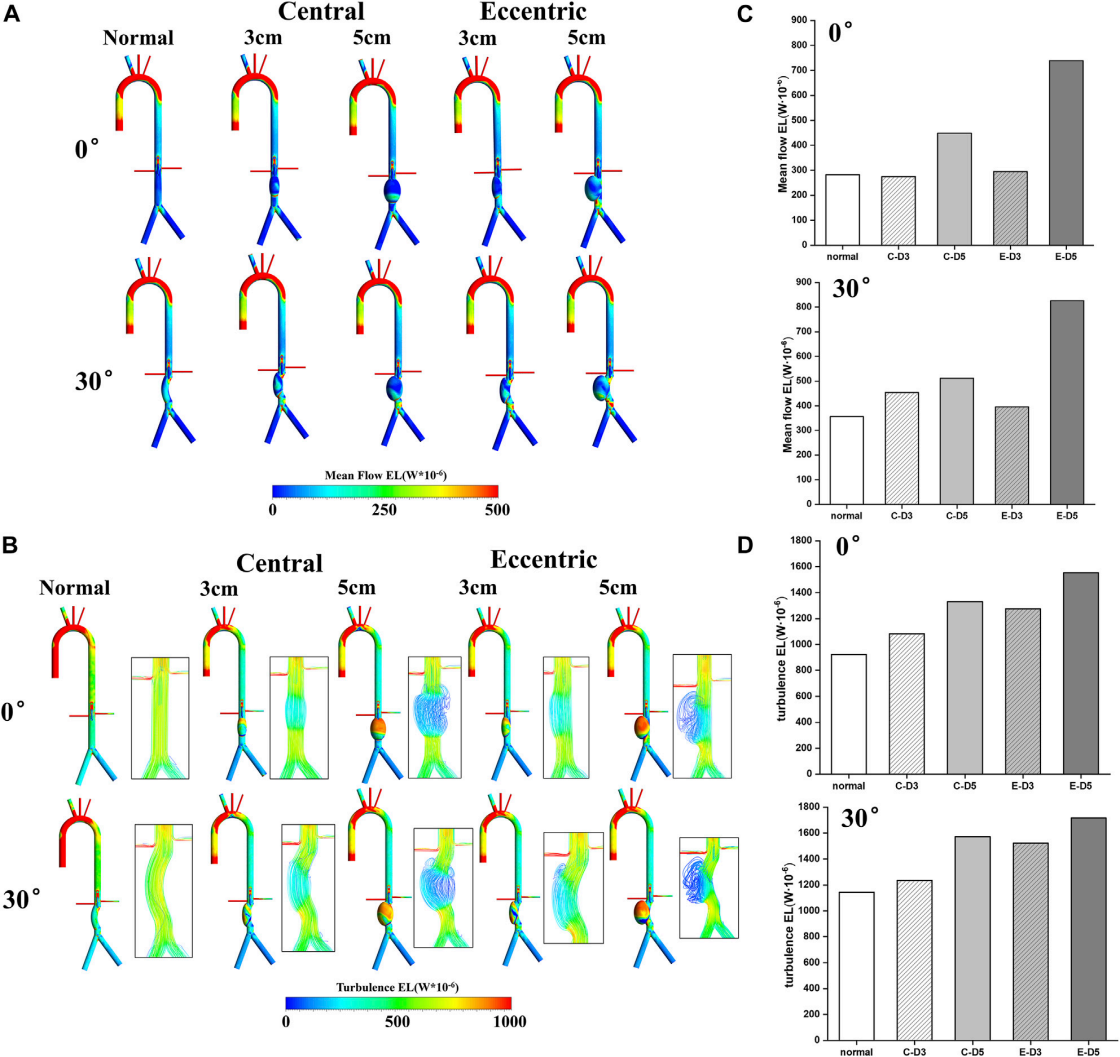
Energy loss: **(A)** Mean flow EL contours; **(B)** turbulence EL contours and flow pattern in the aneurysms; **(C)** the value of mean flow EL in the aneurysms, the C represents central model and the E represents eccentric model; **(D)** the value of turbulence EL in the aneurysms, the C represents central model and the E represents eccentric model.

The mean flow EL mostly occurred near the aneurysm wall, which was similar to TAWSS. By comparing Figure 3A; Figure 5A, it can be found that the distribution of these two metrics was roughly the same, and the high mean flow EL regions were mostly located at the lower part of the aneurysm. In the curvature models and eccentric models, the mean flow EL was also higher at the lateral edge of the aneurysm. Different from the mean flow EL, the turbulence EL mostly occurred in the disturbed flow. As shown in Figure 5B, the flow motion can be observed at the upper part of the aneurysms was slow, vortical and chaotic, and the degree of disorder will exacerbate with the increase of aneurysm diameter. The same phenomenon also occurred in curvature models and eccentric models. Notably, the region with high turbulence EL was highly consistent with the region with disordered flow, indicating the instability of blood flow within the aneurysm, which means a higher risk of rupture.

The value of the mean flow EL and the turbulence EL in aneurysms were collected and shown in Figures 5C, D. It can be found that the increase of aneurysm diameter, curvature, and eccentricity all lead to the increase of the mean flow EL and turbulence EL. Therefore, the eccentric 5 cm model with the bending angle of 30° had the highest mean flow EL and turbulence EL, which were 0.82 W · 10^−3^ and 1.72 W · 10^−3^, respectively. It follows that the diameter, eccentricity and curvature of aneurysms would aggravate the damage and the disturbance of flow in aneurysm, which may play a predictive role in the risk of aneurysm rupture.

## Discussion

Comparing with the normal model, there were more chaotic hemodynamic features within the curvature models and eccentric models. The altered geometries of the aneurysm affected the hemodynamics of the vessel. In previous studies, conventional hemodynamic parameters were mostly included, such as TAWSS, OSI, RRT and ECAP, which are controversial in terms of whether they can consistently and accurately reflect the possible risk of thrombosis and rupture risk of AAA. The region of low TAWSS, high OSI, high RRT and high ECAP values was generally considered to be closely related to the formation of thrombosis (Jiang et al., 2023). However, Lozowy et al. (Lozowy et al., 2017) demonstrated that aneurysmal recirculation zones could have either high or low OSI and there was no correlation between OSI and ILT deposition. Similar results also showed in the study of Zambrano et al. (Zambrano et al., 2022), which believed that the accumulation of ILT occurred in both high and low OSI regions. Therefore, it is significant to seek alternative hemodynamic metrics to predict the risk of thrombosis and aneurysm rupture. Recently, EL has received more attention as a potential predictor of the occurrence of cardiovascular disease. EL can not only quantitatively estimate the quality of congenital heart disease (CHD) surgery (Qian et al., 2010), but also quantify left ventricular loading by using the 4D MRI technique (Barker et al., 2014). Therefore, this metric may have the potential to predict the occurrence of AAA. Qiao et al. (Qiao et al., 2022) showed that aortic aneurysm can lead to a significant loss of blood energy. This study represents the first attempt to use EL evaluate the risk of aneurysm rupture. 10 ideal models were conducted hemodynamic simulation with the same time-varying volumetric flow rate applying at the inlet and the pressure outlet was modulated with 3EWM. In combination with EL and other hemodynamic parameters such as TAWSS, OSI, RRT and ECAP, the effect of the diameter, curvature and eccentricity of AAA on aneurysm rupture and thrombosis have been assessed.

It is common that ILT accumulates within AAA, and whether ILT promotes AAA rupture has been controversial. A layer of ILT may reduce the supply of oxygen to the aortic wall, causing more inflammatory response and making the wall thinner, which makes it more susceptible to further distension or rupture (Vorp et al., 1996; Kazi et al., 2003). In contrast, the formation of a layer of ILT avoids direct exposure of the aortic wall to low and oscillating WSS, which provides a protective effect on the aortic wall (Lasheras, 2007). Through the analysis of hemodynamic parameters in idealized AAA models, it can be found that larger aneurysms may more likely to thrombosis, and the curvature and eccentricity seem to prevent thrombosis. The simulation results of TAWSS and OSI suggested that the distribution of areas with high OSI and low TAWSS was similar in the aneurysm model, and these areas often implied an increased risk of thrombosis, which was similar to the results of Kumar et al. (Kumar et al., 2023). In a recent study, the regions with high TAWSS, low OSI, and low RRT present in the middle and lower aneurysm walls, in where the risk of ILT formation and development was reduced (Zhan et al., 2022). In our study, the low TAWSS area concentrated in the upper part and the lateral edge of the aneurysm, similar to the distribution of areas with high OSI, high RRT and high ECAP, predicting the high risk of thrombosis.

In addition, EL may play a predictive role in the risk of aneurysm rupture. Several studies believed that high OSI region meant the instability of flow patterns, and flow field was in a highly oscillating state, which was related to the rupture of the aneurysm (Zambrano et al., 2016; Chisci et al., 2018). In our study, the region with high turbulence EL was also considered to be the location where the flow field is unstable, which was in consistent with OSI. Furthermore, as a volumetric parameter reflecting the hydraulic loss, EL seems to be a more appropriate metric for the risk of aneurysm rupture. A recent study that explored the pathogenesis and predict lesion development in the idealized aortic aneurysm using the fluid-structure interaction (FSI) method found that there is increased loss of blood energy in aneurysms, which may increase the risk of aneurysm rupture (Qiao et al., 2022). Similar results were confirmed in our study, it can be observed from Figure 5 that, both mean flow EL and turbulent EL were significantly increased in AAA, and the region with high turbulence EL was consistent with disordered flow pattern, indicating the risk of aneurysm rupture.

Moreover, this study also shows that the diameter, eccentricity and curvature of aneurysms would aggravate the damage and the disturbance of flow in aneurysm, which is related to the rupture of aneurysm. It can be observed from the results that, the high mean flow EL and turbulence EL appear in 5 cm models, curvature models and eccentric models, revealing that larger diameter, curvature, and eccentricity seems to be associated with a higher risk of rupture, which is also consistent with previous clinical research (Han et al., 2021; Ozawa et al., 2023; Rastogi et al., 2023).

## Limitation

This study also has some limitations. First, blood is considered as a non-Newtonian fluid, which may influence the accuracy of results in the research. It has been recognized that considering the Newtonian fluid properties of blood does not significantly change the flow field of blood (Perktold et al., 1991) and the correlation with disease (Friedman et al., 1992). Frolov et al. (Frolov et al., 2016) found that the WSS values were similar for both fluids, and the lowest value of the WSS was obtained at the same aneurysm region. Similarly, Newtonian and non-Newtonian fluids have been reported to hardly affect the hemodynamic parameters of the coronary arteries (Kabinejadian and Ghista, 2012; Vimmr et al., 2013). Thus, blood can be considered as a Newtonian fluid for hemodynamic simulation in this study. Second, since most turbulence simulation methods cannot accurately calculate turbulence EL $\varepsilon_{turb}$ , which is a combination of $\varepsilon_{turb,res}$ and $\varepsilon_{turb,\mathrm{mod}}$ by using analytical and simulation methods, $\varepsilon_{turb,\mathrm{mod}}$ was not taken into account because of its low value and less physical significance, and $\varepsilon_{turb,res}$ was included in the analysis of this research in place of turbulence EL $\varepsilon_{turb}$ . Third, the inlet of the computational model was truncated in this study, making it hard to reappear the jet flow of the aortic sinus. In the future, the effect of jet flow of the aortic sinus on hemodynamics should be considered when setting inlet boundary conditions in subsequent studies. Fourth, FSI method is one of important ways to analyze the hemodynamic characteristic, which accounts for the strong interactions between flowing blood and the deforming vessel walls (Philip et al., 2022; Lan et al., 2023). FSI effects should be accounted in the subsequent studies to improve our findings. Moreover, the same flow rate was used for all idealized models. In the future, patient-specific flow rates should be measured in patient-specific models and a sensitivity analysis for boundary conditions should be conducted in the follow-up study. Finally, in this work, the AAA models are idealized and we did not include patient-specific models as well as a more detailed delineation of the ideal model. In the future, more subgroups of different aneurysm diameter, curvature, and eccentricity should be set up in subsequent studies, and a large cohort of patient-specific models should be included to further confirm our findings.

## Conclusion

In summary, through the hemodynamic simulation of idealized AAA models, the diameter, eccentricity and curvature of aneurysms would aggravate the disturbance of flow in aneurysm, which may play a predictive role in the risk of aneurysm rupture. Larger aneurysms may more likely to thrombosis, and the curvature and eccentricity seem to prevent thrombosis. High turbulence EL indicates the instability of blood flow and may be a predictor of the risk of aneurysm rupture. Due to the higher risk of rupture of aneurysms with greater curvature and eccentricity, it may be possible to intervene clinically earlier by hemodynamic prediction compared to the threshold of arterial diameter recommended in clinical guidelines.

## Data Availability

The original contributions presented in the study are included in the article/Supplementary material, further inquiries can be directed to the corresponding authors.

## Abbreviations

3 EWM, 3-element Windkessel model; TAWSS, time-averaged wall shear stress; OSI, oscillatory shear index; RRT, relative residence time; ECAP, endothelial cell activation potential; EL, energy loss; AAA, Abdominal aortic aneurysm; ILT, intraluminal thrombus; CFD, Computational fluid dynamics; BCT, brachiocephalic trunk; LCA, left carotid artery; LSA, left subclavian artery; LRA/RRA, right/left renal artery; CA, celiac artery; SMA, superior mesenteric artery; LIA/RIA, left/right iliac artery; CHD, congenital heart disease.
